# Nitratotris(triphenyl­phosphine)copper(I) methanol solvate

**DOI:** 10.1107/S1600536809004620

**Published:** 2009-02-13

**Authors:** Gideon Steyl

**Affiliations:** a Department of Chemistry, University of the Free State, Bloemfontein 9300, South Africa

## Abstract

The title compound, [Cu(NO_3_)(C_18_H_15_P)_3_]·CH_3_OH, is a methanol solvate derivative of nitratotris(triphenyl­phos­phine)copper(I). The complex crystallizes with three triphenyl­phosphine ligands coordinated to the copper centre, with an O—H⋯O hydrogen bond observed between the nitrate ligand and the methanol solvent mol­ecule. The coordination around the Cu^I^ centre is distorted tetrahedral.

## Related literature

The title compound is structurally related to the ethanol solvate derivative (Dyason *et al.*, 1986 ▶). For related diketonato complexes, see: Hill & Steyl (2008 ▶); Steyl & Roodt (2006 ▶); Steyl (2007 ▶); Steyl & Hill (2009 ▶). For general background, see: Roodt *et al.* (2003 ▶); Crous *et al.* (2005 ▶).
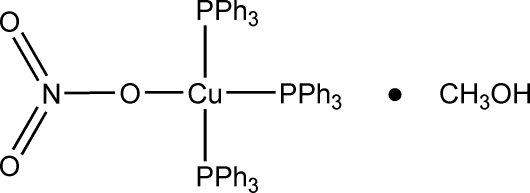


## Experimental

### 

#### Crystal data

[Cu(NO_3_)(C_18_H_15_P)_3_]·CH_4_O
*M* *_r_* = 944.40
Monoclinic, 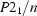

*a* = 14.016 (2) Å
*b* = 23.015 (3) Å
*c* = 14.765 (2) Å
β = 92.569 (1)°
*V* = 4758.08 (11) Å^3^
*Z* = 4
Mo *K*α radiation
μ = 0.61 mm^−1^
*T* = 143 K
0.16 × 0.14 × 0.12 mm

#### Data collection

Bruker APEXII 4K CCD area-detector diffractometer
Absorption correction: multi-scan (*SADABS*; Sheldrick, 1999 ▶) *T*_min_ = 0.909, *T*_max_ = 0.931
60001 measured reflections
10386 independent reflections
7282 reflections with *I* > 2σ(*I*)
*R*_int_ = 0.045

#### Refinement

*R*[*F* ^2^ > 2σ(*F* ^2^)] = 0.042
*wR*(*F* ^2^) = 0.125
*S* = 1.07
10386 reflections
579 parameters
H-atom parameters constrained
Δρ_max_ = 0.70 e Å^−3^
Δρ_min_ = −0.49 e Å^−3^

### 

Data collection: *APEX2* (Bruker, 2005 ▶); cell refinement: *SAINT-Plus* (Bruker, 2004 ▶); data reduction: *SAINT-Plus*; program(s) used to solve structure: *SHELXS97* (Sheldrick, 2008 ▶); program(s) used to refine structure: *SHELXL97* (Sheldrick, 2008 ▶); molecular graphics: *DIAMOND* (Brandenburg & Putz, 2006 ▶); software used to prepare material for publication: *SHELXL97*.

## Supplementary Material

Crystal structure: contains datablocks I, global. DOI: 10.1107/S1600536809004620/lx2086sup1.cif

Structure factors: contains datablocks I. DOI: 10.1107/S1600536809004620/lx2086Isup2.hkl

Additional supplementary materials:  crystallographic information; 3D view; checkCIF report

## Figures and Tables

**Table:** 

Cu—O1	2.1503 (18)
Cu—P2	2.3070 (7)
Cu—P3	2.3321 (6)
Cu—P1	2.3397 (6)

**Table:** 

O1—Cu—P2	109.78 (5)
O1—Cu—P3	95.11 (6)
P2—Cu—P3	113.80 (2)
O1—Cu—P1	98.21 (5)
P2—Cu—P1	121.70 (2)
P3—Cu—P1	113.24 (2)

**Table 2:** Hydrogen-bond geometry (Å, °)

*D*—H⋯*A*	*D*—H	H⋯*A*	*D*⋯*A*	*D*—H⋯*A*
O01—H01⋯O2	0.84	2.03	2.835 (3)	159

**Fig. 1.**
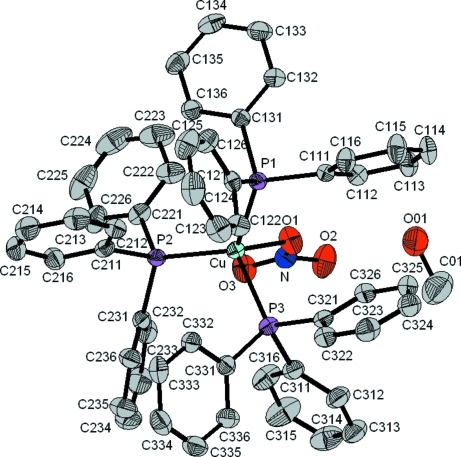
: Representation of the title compound (I), showing the numbering scheme and displacement ellipsoids (50 % probability). For the carbon rings on the triphenylphosphine ligands, the first digit refers to phosphorous number, second digit to the ring number and third digit to atom in the ring. Hydrogen atoms omitted for clarity.

## supplementary crystallographic information

### Comment

The title compound, (I), is an example of a methanol solvate of a previously published ethanol solvate complex (Dyason *et al.*, 1986). Both complexes crystallize in the *P*2_1_/*n* space group with similar cell lengths and angles. The inclusion of a methanol solvate compared to a ethanol solvate molecule is best illustrated in the cell volume increase from 4758 to 4909 Å^3^.

The Cu—P bond distances differ from each other in the title compound, Table 1, with one triphenyl phosphine moiety (P2) being slightly closer to the copper metal centre compared to the remaining ligands. An intermolecular hydrogen bond is observed between the solvate molecule and the nitrato moiety coordinated to the copper centre, Table 2.

The ethanol complex (Dyason *et al.*, 1986) is closely related to the title compound with only minor differences in bond lengths and angles. A similar hydrogen bond is observed between the solvate molecule and the nitrato moiety in each of these compounds. The O···O bond distance increases from 2.772 to 2.835 Å from the ethanol to the methanol solvate system.

### Experimental

The title complex was synthesised by recrystallizing the [Cu(PPh_3_)_2_NO_3_] complex from hot methanol. On slow evaporation of the solvent; yellow crystals suitable for X-Ray crystallography was obtained.

### Refinement

H atoms were positioned geometrically and refined using a riding model, with C—H = 0.95 (C aromatic) and 0.99 (methyl) Å and *U*_iso_(H) = 1.2 times *U*_eq_.

### Crystal data

**Table:**

[Cu(NO_3_)(C_18_H_15_P)_3_]·CH_4_O	*F*(000) = 1968
*M**_r_* = 944.40	*D*_x_ = 1.318 Mg m^−^^3^
Monoclinic, *P*2_1_/*n*	Mo *K*α radiation, λ = 0.71073 Å
Hall symbol: -P 2yn	Cell parameters from 9106 reflections
*a* = 14.0160 (2) Å	θ = 2.3–28.3°
*b* = 23.0150 (3) Å	µ = 0.61 mm^−^^1^
*c* = 14.7650 (2) Å	*T* = 143 K
β = 92.569 (1)°	Cuboid, yellow
*V* = 4758.08 (11) Å^3^	0.16 × 0.14 × 0.12 mm
*Z* = 4	

### Data collection

**Table:**

Bruker APEXII 4K CCD area-detector diffractometer	10386 independent reflections
Radiation source: fine-focus sealed tube	7282 reflections with *I* > 2σ(*I*)
graphite	*R*_int_ = 0.045
Detector resolution: 512 x 512 pixels mm^-1^	θ_max_ = 27.0°, θ_min_ = 2.2°
φ and ω scans	*h* = −17→17
Absorption correction: multi-scan (*SADABS*; Sheldrick, 1999)	*k* = −29→29
*T*_min_ = 0.909, *T*_max_ = 0.931	*l* = −18→18
60001 measured reflections	

### Refinement

**Table:**

Refinement on *F*^2^	Primary atom site location: structure-invariant direct methods
Least-squares matrix: full	Secondary atom site location: difference Fourier map
*R*[*F*^2^ > 2σ(*F*^2^)] = 0.042	Hydrogen site location: inferred from neighbouring sites
*wR*(*F*^2^) = 0.125	H-atom parameters constrained
*S* = 1.07	*w* = 1/[σ^2^(*F*_o_^2^) + (0.0652*P*)^2^ + 1.3303*P*] where *P* = (*F*_o_^2^ + 2*F*_c_^2^)/3
10386 reflections	(Δ/σ)_max_ < 0.001
579 parameters	Δρ_max_ = 0.70 e Å^−^^3^
0 restraints	Δρ_min_ = −0.49 e Å^−^^3^

### Special details

**Table:**

Geometry. All s.u.'s (except the s.u. in the dihedral angle between two l.s. planes) are estimated using the full covariance matrix. The cell s.u.'s are taken into account individually in the estimation of s.u.'s in distances, angles and torsion angles; correlations between s.u.'s in cell parameters are only used when they are defined by crystal symmetry. An approximate (isotropic) treatment of cell s.u.'s is used for estimating s.u.'s involving l.s. planes.
Refinement. Refinement of F^2^ against ALL reflections. The weighted R-factor wR and goodness of fit S are based on F^2^, conventional R-factors R are based on F, with F set to zero for negative F^2^. The threshold expression of F^2^ > 2σ(F^2^) is used only for calculating R-factors(gt) etc. and is not relevant to the choice of reflections for refinement. R-factors based on F^2^ are statistically about twice as large as those based on F, and R- factors based on ALL data will be even larger.

### Fractional atomic coordinates and isotropic or equivalent isotropic displacement parameters (Å^2^)

**Table:**

	*x*	*y*	*z*	*U*_iso_*/*U*_eq_	
Cu	0.648980 (19)	0.639638 (12)	0.721924 (18)	0.02506 (9)	
P1	0.68719 (4)	0.66836 (3)	0.57592 (4)	0.02327 (14)	
P2	0.49525 (4)	0.64599 (3)	0.77073 (4)	0.02451 (14)	
P3	0.76607 (4)	0.66405 (3)	0.83260 (4)	0.02716 (15)	
O1	0.69049 (14)	0.54993 (8)	0.71328 (13)	0.0426 (5)	
O2	0.70267 (14)	0.46327 (8)	0.76821 (16)	0.0545 (6)	
O3	0.58208 (14)	0.51654 (9)	0.79920 (14)	0.0476 (5)	
N	0.65747 (16)	0.50981 (9)	0.76134 (15)	0.0339 (5)	
C111	0.80233 (16)	0.64237 (10)	0.53885 (15)	0.0242 (5)	
C124	0.7058 (2)	0.86739 (12)	0.5420 (2)	0.0513 (8)	
H124	0.7091	0.9085	0.5366	0.062*	
C224	0.2713 (3)	0.52621 (15)	0.6295 (3)	0.0669 (11)	
H224	0.2253	0.5025	0.5982	0.080*	
C316	0.7201 (2)	0.58002 (13)	0.9585 (2)	0.0479 (7)	
H316	0.6561	0.5870	0.9373	0.057*	
C231	0.48468 (16)	0.63742 (11)	0.89305 (16)	0.0278 (5)	
C321	0.88416 (17)	0.67722 (11)	0.78815 (16)	0.0296 (5)	
C131	0.60777 (17)	0.64440 (10)	0.48089 (15)	0.0255 (5)	
C115	0.94606 (19)	0.65381 (13)	0.4577 (2)	0.0442 (7)	
H115	0.9884	0.6780	0.4262	0.053*	
C311	0.79398 (18)	0.60965 (11)	0.92032 (16)	0.0318 (6)	
C332	0.69771 (17)	0.77617 (11)	0.85457 (17)	0.0324 (6)	
H332	0.6763	0.7723	0.7929	0.039*	
C112	0.82424 (17)	0.58383 (11)	0.55058 (16)	0.0294 (5)	
H112	0.7827	0.5595	0.5827	0.035*	
C211	0.43902 (16)	0.71734 (10)	0.75056 (15)	0.0269 (5)	
C331	0.74432 (16)	0.72969 (11)	0.89767 (16)	0.0283 (5)	
C132	0.64211 (19)	0.61962 (12)	0.40217 (16)	0.0338 (6)	
H132	0.7089	0.6154	0.3960	0.041*	
C221	0.40535 (18)	0.59655 (11)	0.72002 (17)	0.0319 (6)	
C216	0.35266 (18)	0.73328 (12)	0.78759 (18)	0.0370 (6)	
H216	0.3226	0.7075	0.8276	0.044*	
C122	0.74845 (19)	0.77996 (11)	0.62032 (18)	0.0349 (6)	
H122	0.7824	0.7610	0.6690	0.042*	
C312	0.8876 (2)	0.59846 (13)	0.95272 (18)	0.0411 (7)	
H312	0.9393	0.6186	0.9276	0.049*	
C225	0.2474 (2)	0.55510 (14)	0.7067 (3)	0.0621 (10)	
H225	0.1853	0.5509	0.7290	0.075*	
C212	0.48208 (18)	0.75662 (11)	0.69410 (16)	0.0316 (6)	
H212	0.5410	0.7468	0.6687	0.038*	
C121	0.69420 (16)	0.74698 (10)	0.55818 (16)	0.0267 (5)	
C326	0.92508 (18)	0.63211 (13)	0.73989 (17)	0.0368 (6)	
H326	0.8916	0.5965	0.7311	0.044*	
C116	0.86462 (18)	0.67765 (11)	0.49296 (18)	0.0347 (6)	
H116	0.8515	0.7179	0.4857	0.042*	
C113	0.90613 (18)	0.56075 (11)	0.51586 (18)	0.0356 (6)	
H113	0.9210	0.5209	0.5250	0.043*	
C133	0.5789 (2)	0.60125 (13)	0.33334 (18)	0.0421 (7)	
H133	0.6028	0.5844	0.2801	0.050*	
C222	0.4288 (2)	0.56572 (11)	0.64328 (18)	0.0419 (7)	
H222	0.4916	0.5681	0.6219	0.050*	
C125	0.6523 (2)	0.83553 (13)	0.4786 (2)	0.0507 (8)	
H125	0.6198	0.8548	0.4294	0.061*	
C136	0.50933 (18)	0.65097 (12)	0.48728 (17)	0.0340 (6)	
H136	0.4847	0.6684	0.5397	0.041*	
C114	0.96614 (19)	0.59556 (12)	0.4680 (2)	0.0414 (7)	
H114	1.0211	0.5794	0.4422	0.050*	
C322	0.93414 (18)	0.72892 (12)	0.79913 (17)	0.0353 (6)	
H322	0.9072	0.7599	0.8319	0.042*	
C324	1.0635 (2)	0.69100 (16)	0.71702 (19)	0.0497 (8)	
H324	1.1252	0.6955	0.6937	0.060*	
C323	1.02253 (19)	0.73598 (14)	0.76306 (19)	0.0431 (7)	
H323	1.0552	0.7720	0.7700	0.052*	
C215	0.3105 (2)	0.78637 (13)	0.7665 (2)	0.0449 (7)	
H215	0.2512	0.7964	0.7911	0.054*	
C134	0.4815 (2)	0.60707 (12)	0.34115 (18)	0.0409 (7)	
H134	0.4385	0.5938	0.2940	0.049*	
C135	0.44731 (19)	0.63221 (13)	0.41747 (19)	0.0409 (7)	
H135	0.3804	0.6368	0.4226	0.049*	
C334	0.7111 (2)	0.83285 (13)	0.9899 (2)	0.0438 (7)	
H334	0.6995	0.8679	1.0217	0.053*	
C214	0.3545 (2)	0.82457 (12)	0.7097 (2)	0.0464 (7)	
H214	0.3255	0.8609	0.6951	0.056*	
C333	0.68171 (18)	0.82795 (12)	0.8992 (2)	0.0395 (6)	
H333	0.6512	0.8595	0.8683	0.047*	
C213	0.4401 (2)	0.81017 (12)	0.67419 (19)	0.0426 (7)	
H213	0.4708	0.8368	0.6359	0.051*	
C126	0.64584 (19)	0.77566 (12)	0.48651 (19)	0.0391 (6)	
H126	0.6084	0.7541	0.4431	0.047*	
C123	0.7541 (2)	0.83983 (12)	0.6128 (2)	0.0448 (7)	
H123	0.7910	0.8617	0.6563	0.054*	
C336	0.77430 (19)	0.73630 (12)	0.98842 (17)	0.0375 (6)	
H336	0.8070	0.7054	1.0192	0.045*	
C226	0.31446 (19)	0.59069 (13)	0.7526 (2)	0.0454 (7)	
H226	0.2979	0.6108	0.8058	0.054*	
C325	1.0142 (2)	0.63923 (14)	0.7049 (2)	0.0459 (7)	
H325	1.0418	0.6084	0.6723	0.055*	
C313	0.9051 (2)	0.55805 (14)	1.02142 (19)	0.0483 (8)	
H313	0.9688	0.5505	1.0428	0.058*	
C232	0.46013 (18)	0.58494 (12)	0.93197 (18)	0.0381 (6)	
H232	0.4420	0.5529	0.8944	0.046*	
C236	0.51236 (19)	0.68329 (13)	0.94916 (17)	0.0381 (6)	
H236	0.5305	0.7192	0.9231	0.046*	
C223	0.3604 (3)	0.53138 (13)	0.5977 (2)	0.0601 (9)	
H223	0.3761	0.5115	0.5440	0.072*	
C235	0.5140 (2)	0.67760 (16)	1.0430 (2)	0.0543 (8)	
H235	0.5321	0.7095	1.0808	0.065*	
C335	0.7569 (2)	0.78753 (13)	1.03417 (19)	0.0440 (7)	
H335	0.7766	0.7913	1.0963	0.053*	
C233	0.4620 (2)	0.57908 (15)	1.0260 (2)	0.0501 (8)	
H233	0.4446	0.5432	1.0524	0.060*	
C314	0.8304 (3)	0.52900 (14)	1.0585 (2)	0.0565 (9)	
H314	0.8425	0.5014	1.1054	0.068*	
C234	0.4890 (2)	0.62522 (18)	1.0805 (2)	0.0587 (9)	
H234	0.4905	0.6209	1.1445	0.070*	
C315	0.7387 (3)	0.53994 (15)	1.0278 (2)	0.0629 (10)	
H315	0.6872	0.5201	1.0538	0.075*	
O01	0.88218 (16)	0.46711 (12)	0.68724 (15)	0.0648 (6)	
H01	0.8262	0.4736	0.7028	0.097*	
C01	0.9416 (3)	0.4603 (2)	0.7637 (3)	0.0824 (12)	
H01A	1.0077	0.4558	0.7458	0.124*	
H01B	0.9225	0.4257	0.7970	0.124*	
H01C	0.9369	0.4946	0.8027	0.124*	

### Atomic displacement parameters (Å^2^)

**Table:**

	*U*^11^	*U*^22^	*U*^33^	*U*^12^	*U*^13^	*U*^23^
Cu	0.02526 (16)	0.02543 (16)	0.02440 (16)	−0.00043 (12)	0.00013 (12)	0.00087 (11)
P1	0.0239 (3)	0.0232 (3)	0.0227 (3)	0.0005 (2)	0.0004 (2)	0.0001 (2)
P2	0.0247 (3)	0.0264 (3)	0.0224 (3)	−0.0005 (2)	0.0009 (2)	0.0006 (2)
P3	0.0256 (3)	0.0297 (3)	0.0258 (3)	−0.0007 (3)	−0.0028 (2)	0.0002 (3)
O1	0.0506 (12)	0.0282 (10)	0.0504 (12)	0.0031 (9)	0.0178 (9)	0.0087 (9)
O2	0.0473 (12)	0.0289 (10)	0.0873 (16)	0.0077 (9)	0.0029 (11)	0.0159 (10)
O3	0.0456 (12)	0.0458 (12)	0.0526 (12)	−0.0012 (9)	0.0158 (10)	0.0115 (10)
N	0.0362 (12)	0.0271 (12)	0.0382 (12)	−0.0005 (10)	0.0001 (10)	0.0029 (9)
C111	0.0233 (11)	0.0271 (12)	0.0223 (11)	0.0010 (10)	0.0009 (9)	−0.0017 (9)
C124	0.0514 (19)	0.0226 (14)	0.081 (2)	0.0014 (13)	0.0130 (17)	0.0052 (14)
C224	0.074 (3)	0.0410 (19)	0.081 (3)	−0.0160 (18)	−0.042 (2)	0.0059 (18)
C316	0.0402 (16)	0.0503 (18)	0.0519 (18)	−0.0091 (14)	−0.0103 (14)	0.0147 (14)
C231	0.0221 (12)	0.0379 (14)	0.0236 (12)	0.0016 (10)	0.0037 (9)	0.0042 (10)
C321	0.0255 (12)	0.0387 (14)	0.0241 (12)	0.0005 (11)	−0.0039 (10)	−0.0006 (10)
C131	0.0304 (13)	0.0237 (12)	0.0226 (11)	0.0004 (10)	0.0018 (10)	0.0028 (9)
C115	0.0307 (14)	0.0436 (17)	0.0596 (19)	0.0028 (12)	0.0181 (13)	0.0124 (14)
C311	0.0342 (14)	0.0318 (14)	0.0290 (13)	0.0015 (11)	−0.0039 (11)	0.0001 (10)
C332	0.0268 (13)	0.0389 (15)	0.0316 (13)	−0.0005 (11)	0.0012 (10)	−0.0008 (11)
C112	0.0296 (13)	0.0280 (13)	0.0307 (13)	−0.0017 (10)	0.0014 (10)	0.0013 (10)
C211	0.0265 (12)	0.0276 (13)	0.0259 (12)	−0.0008 (10)	−0.0049 (10)	−0.0015 (10)
C331	0.0238 (12)	0.0308 (13)	0.0304 (13)	−0.0025 (10)	0.0013 (10)	−0.0020 (10)
C132	0.0349 (14)	0.0385 (14)	0.0277 (13)	0.0023 (12)	−0.0014 (11)	−0.0038 (11)
C221	0.0328 (14)	0.0260 (13)	0.0363 (14)	−0.0033 (11)	−0.0061 (11)	0.0029 (11)
C216	0.0370 (15)	0.0365 (15)	0.0379 (15)	0.0036 (12)	0.0048 (12)	−0.0038 (12)
C122	0.0411 (15)	0.0305 (14)	0.0332 (14)	−0.0041 (12)	0.0028 (11)	0.0002 (11)
C312	0.0375 (15)	0.0481 (17)	0.0367 (15)	0.0036 (13)	−0.0076 (12)	0.0053 (12)
C225	0.0368 (17)	0.0452 (19)	0.102 (3)	−0.0100 (15)	−0.0191 (18)	0.0133 (19)
C212	0.0348 (14)	0.0311 (14)	0.0290 (13)	−0.0008 (11)	0.0015 (11)	−0.0016 (10)
C121	0.0274 (12)	0.0239 (12)	0.0294 (12)	0.0010 (10)	0.0075 (10)	0.0013 (10)
C326	0.0319 (14)	0.0475 (17)	0.0307 (14)	0.0022 (12)	−0.0014 (11)	−0.0048 (12)
C116	0.0304 (13)	0.0288 (13)	0.0455 (15)	0.0030 (11)	0.0057 (11)	0.0041 (11)
C113	0.0332 (14)	0.0277 (13)	0.0461 (15)	0.0071 (11)	0.0041 (12)	−0.0023 (11)
C133	0.0520 (18)	0.0449 (17)	0.0288 (14)	0.0029 (14)	−0.0031 (12)	−0.0045 (12)
C222	0.0574 (18)	0.0295 (14)	0.0379 (15)	−0.0071 (13)	−0.0070 (13)	0.0003 (11)
C125	0.0468 (17)	0.0388 (17)	0.066 (2)	0.0064 (14)	−0.0029 (15)	0.0189 (15)
C136	0.0281 (13)	0.0454 (16)	0.0286 (13)	−0.0024 (11)	0.0021 (10)	−0.0002 (11)
C114	0.0290 (14)	0.0411 (16)	0.0553 (18)	0.0080 (12)	0.0130 (13)	−0.0032 (13)
C322	0.0339 (14)	0.0385 (15)	0.0333 (14)	−0.0008 (12)	−0.0015 (11)	−0.0028 (11)
C324	0.0304 (15)	0.081 (2)	0.0378 (16)	−0.0006 (15)	0.0052 (12)	0.0010 (16)
C323	0.0325 (15)	0.0550 (19)	0.0416 (16)	−0.0104 (13)	0.0015 (12)	0.0056 (14)
C215	0.0403 (16)	0.0460 (17)	0.0483 (17)	0.0149 (14)	−0.0002 (13)	−0.0113 (14)
C134	0.0425 (16)	0.0422 (16)	0.0365 (15)	−0.0084 (13)	−0.0146 (12)	0.0025 (12)
C135	0.0269 (14)	0.0537 (18)	0.0414 (16)	−0.0059 (12)	−0.0053 (12)	0.0072 (13)
C334	0.0380 (15)	0.0426 (17)	0.0515 (18)	−0.0018 (13)	0.0099 (13)	−0.0162 (14)
C214	0.0579 (19)	0.0321 (15)	0.0480 (17)	0.0135 (14)	−0.0103 (15)	−0.0036 (13)
C333	0.0304 (14)	0.0352 (15)	0.0535 (17)	0.0027 (12)	0.0098 (12)	−0.0006 (13)
C213	0.0579 (19)	0.0305 (14)	0.0386 (15)	0.0007 (13)	−0.0061 (14)	0.0030 (12)
C126	0.0375 (15)	0.0367 (15)	0.0427 (16)	0.0015 (12)	−0.0037 (12)	0.0059 (12)
C123	0.0494 (17)	0.0307 (15)	0.0547 (18)	−0.0079 (13)	0.0071 (14)	−0.0060 (13)
C336	0.0403 (15)	0.0431 (16)	0.0289 (13)	−0.0021 (12)	−0.0001 (11)	−0.0022 (11)
C226	0.0325 (15)	0.0419 (16)	0.0614 (19)	−0.0019 (13)	−0.0022 (14)	−0.0007 (14)
C325	0.0341 (15)	0.065 (2)	0.0385 (16)	0.0145 (14)	−0.0016 (12)	−0.0098 (14)
C313	0.0501 (18)	0.0501 (18)	0.0433 (16)	0.0129 (15)	−0.0130 (14)	0.0027 (14)
C232	0.0334 (14)	0.0414 (16)	0.0402 (15)	0.0023 (12)	0.0087 (12)	0.0108 (12)
C236	0.0392 (15)	0.0480 (17)	0.0274 (13)	−0.0018 (13)	0.0040 (11)	−0.0033 (12)
C223	0.090 (3)	0.0376 (17)	0.0505 (19)	−0.0110 (17)	−0.0200 (19)	−0.0087 (14)
C235	0.0549 (19)	0.078 (2)	0.0306 (15)	0.0007 (17)	0.0022 (14)	−0.0108 (15)
C335	0.0457 (17)	0.0538 (18)	0.0325 (15)	−0.0031 (14)	0.0013 (13)	−0.0116 (13)
C233	0.0452 (17)	0.065 (2)	0.0409 (17)	0.0058 (15)	0.0136 (14)	0.0273 (16)
C314	0.079 (2)	0.0412 (18)	0.0476 (18)	−0.0019 (17)	−0.0165 (17)	0.0132 (14)
C234	0.0519 (19)	0.097 (3)	0.0274 (15)	0.0098 (19)	0.0061 (14)	0.0106 (17)
C315	0.060 (2)	0.061 (2)	0.066 (2)	−0.0217 (17)	−0.0134 (17)	0.0295 (18)
O01	0.0563 (14)	0.0808 (18)	0.0577 (14)	0.0180 (14)	0.0077 (11)	0.0099 (12)
C01	0.075 (3)	0.083 (3)	0.088 (3)	0.018 (2)	−0.017 (2)	0.005 (2)

### Geometric parameters (Å, °)

**Table:**

Cu—O1	2.1503 (18)	C121—C126	1.397 (3)
Cu—P2	2.3070 (7)	C326—C325	1.383 (4)
Cu—P3	2.3321 (6)	C326—H326	0.9500
Cu—P1	2.3397 (6)	C116—H116	0.9500
P1—C111	1.828 (2)	C113—C114	1.379 (4)
P1—C121	1.832 (2)	C113—H113	0.9500
P1—C131	1.836 (2)	C133—C134	1.382 (4)
P2—C231	1.829 (2)	C133—H133	0.9500
P2—C221	1.833 (2)	C222—C223	1.392 (4)
P2—C211	1.840 (2)	C222—H222	0.9500
P3—C331	1.823 (2)	C125—C126	1.386 (4)
P3—C311	1.831 (3)	C125—H125	0.9500
P3—C321	1.833 (3)	C136—C135	1.387 (4)
O1—N	1.265 (3)	C136—H136	0.9500
O2—N	1.246 (3)	C114—H114	0.9500
O3—N	1.227 (3)	C322—C323	1.380 (4)
C111—C116	1.391 (3)	C322—H322	0.9500
C111—C112	1.391 (3)	C324—C323	1.378 (4)
C124—C123	1.375 (4)	C324—C325	1.385 (4)
C124—C125	1.383 (5)	C324—H324	0.9500
C124—H124	0.9500	C323—H323	0.9500
C224—C223	1.359 (5)	C215—C214	1.380 (4)
C224—C225	1.374 (5)	C215—H215	0.9500
C224—H224	0.9500	C134—C135	1.372 (4)
C316—C311	1.381 (4)	C134—H134	0.9500
C316—C315	1.393 (4)	C135—H135	0.9500
C316—H316	0.9500	C334—C335	1.374 (4)
C231—C236	1.387 (4)	C334—C333	1.388 (4)
C231—C232	1.387 (3)	C334—H334	0.9500
C321—C322	1.387 (4)	C214—C213	1.371 (4)
C321—C326	1.397 (4)	C214—H214	0.9500
C131—C136	1.395 (3)	C333—H333	0.9500
C131—C132	1.399 (3)	C213—H213	0.9500
C115—C114	1.377 (4)	C126—H126	0.9500
C115—C116	1.388 (4)	C123—H123	0.9500
C115—H115	0.9500	C336—C335	1.386 (4)
C311—C312	1.400 (3)	C336—H336	0.9500
C332—C333	1.385 (4)	C226—H226	0.9500
C332—C331	1.392 (3)	C325—H325	0.9500
C332—H332	0.9500	C313—C314	1.377 (5)
C112—C113	1.384 (3)	C313—H313	0.9500
C112—H112	0.9500	C232—C233	1.394 (4)
C211—C212	1.386 (3)	C232—H232	0.9500
C211—C216	1.399 (3)	C236—C235	1.391 (4)
C331—C336	1.394 (3)	C236—H236	0.9500
C132—C133	1.384 (4)	C223—H223	0.9500
C132—H132	0.9500	C235—C234	1.378 (5)
C221—C226	1.388 (4)	C235—H235	0.9500
C221—C222	1.389 (4)	C335—H335	0.9500
C216—C215	1.387 (4)	C233—C234	1.375 (5)
C216—H216	0.9500	C233—H233	0.9500
C122—C123	1.385 (4)	C314—C315	1.367 (5)
C122—C121	1.391 (3)	C314—H314	0.9500
C122—H122	0.9500	C234—H234	0.9500
C312—C313	1.390 (4)	C315—H315	0.9500
C312—H312	0.9500	O01—C01	1.382 (4)
C225—C226	1.398 (4)	O01—H01	0.8400
C225—H225	0.9500	C01—H01A	0.9800
C212—C213	1.391 (4)	C01—H01B	0.9800
C212—H212	0.9500	C01—H01C	0.9800
			
O1—Cu—P2	109.78 (5)	C112—C113—H113	119.9
O1—Cu—P3	95.11 (6)	C134—C133—C132	120.7 (3)
P2—Cu—P3	113.80 (2)	C134—C133—H133	119.6
O1—Cu—P1	98.21 (5)	C132—C133—H133	119.6
P2—Cu—P1	121.70 (2)	C221—C222—C223	120.1 (3)
P3—Cu—P1	113.24 (2)	C221—C222—H222	119.9
C111—P1—C121	103.11 (11)	C223—C222—H222	119.9
C111—P1—C131	100.72 (11)	C124—C125—C126	120.4 (3)
C121—P1—C131	102.82 (10)	C124—C125—H125	119.8
C111—P1—Cu	115.16 (8)	C126—C125—H125	119.8
C121—P1—Cu	115.26 (8)	C135—C136—C131	120.3 (2)
C131—P1—Cu	117.59 (8)	C135—C136—H136	119.8
C231—P2—C221	104.58 (12)	C131—C136—H136	119.8
C231—P2—C211	101.71 (11)	C115—C114—C113	119.7 (2)
C221—P2—C211	101.91 (11)	C115—C114—H114	120.1
C231—P2—Cu	114.81 (8)	C113—C114—H114	120.1
C221—P2—Cu	117.89 (9)	C323—C322—C321	120.9 (3)
C211—P2—Cu	113.91 (8)	C323—C322—H322	119.6
C331—P3—C311	103.25 (11)	C321—C322—H322	119.6
C331—P3—C321	103.10 (11)	C323—C324—C325	119.6 (3)
C311—P3—C321	101.46 (11)	C323—C324—H324	120.2
C331—P3—Cu	116.09 (8)	C325—C324—H324	120.2
C311—P3—Cu	116.89 (8)	C324—C323—C322	120.2 (3)
C321—P3—Cu	114.04 (8)	C324—C323—H323	119.9
N—O1—Cu	124.12 (16)	C322—C323—H323	119.9
O3—N—O2	121.1 (2)	C214—C215—C216	120.0 (3)
O3—N—O1	120.5 (2)	C214—C215—H215	120.0
O2—N—O1	118.4 (2)	C216—C215—H215	120.0
C116—C111—C112	119.1 (2)	C135—C134—C133	119.5 (2)
C116—C111—P1	122.47 (18)	C135—C134—H134	120.3
C112—C111—P1	118.21 (18)	C133—C134—H134	120.3
C123—C124—C125	120.2 (3)	C134—C135—C136	120.7 (3)
C123—C124—H124	119.9	C134—C135—H135	119.6
C125—C124—H124	119.9	C136—C135—H135	119.6
C223—C224—C225	120.4 (3)	C335—C334—C333	120.6 (3)
C223—C224—H224	119.8	C335—C334—H334	119.7
C225—C224—H224	119.8	C333—C334—H334	119.7
C311—C316—C315	120.5 (3)	C213—C214—C215	120.1 (3)
C311—C316—H316	119.8	C213—C214—H214	120.0
C315—C316—H316	119.8	C215—C214—H214	120.0
C236—C231—C232	118.9 (2)	C332—C333—C334	118.8 (3)
C236—C231—P2	118.35 (19)	C332—C333—H333	120.6
C232—C231—P2	122.4 (2)	C334—C333—H333	120.6
C322—C321—C326	118.7 (2)	C214—C213—C212	120.1 (3)
C322—C321—P3	124.0 (2)	C214—C213—H213	119.9
C326—C321—P3	117.2 (2)	C212—C213—H213	119.9
C136—C131—C132	118.6 (2)	C125—C126—C121	120.1 (3)
C136—C131—P1	118.90 (18)	C125—C126—H126	119.9
C132—C131—P1	122.53 (18)	C121—C126—H126	119.9
C114—C115—C116	120.7 (3)	C124—C123—C122	119.5 (3)
C114—C115—H115	119.6	C124—C123—H123	120.3
C116—C115—H115	119.6	C122—C123—H123	120.3
C316—C311—C312	118.5 (2)	C335—C336—C331	120.6 (3)
C316—C311—P3	119.08 (19)	C335—C336—H336	119.7
C312—C311—P3	122.3 (2)	C331—C336—H336	119.7
C333—C332—C331	121.7 (2)	C221—C226—C225	119.8 (3)
C333—C332—H332	119.1	C221—C226—H226	120.1
C331—C332—H332	119.1	C225—C226—H226	120.1
C113—C112—C111	120.5 (2)	C326—C325—C324	120.5 (3)
C113—C112—H112	119.7	C326—C325—H325	119.8
C111—C112—H112	119.7	C324—C325—H325	119.8
C212—C211—C216	118.1 (2)	C314—C313—C312	120.1 (3)
C212—C211—P2	118.98 (18)	C314—C313—H313	119.9
C216—C211—P2	122.85 (19)	C312—C313—H313	119.9
C332—C331—C336	118.1 (2)	C231—C232—C233	120.2 (3)
C332—C331—P3	118.80 (18)	C231—C232—H232	119.9
C336—C331—P3	123.1 (2)	C233—C232—H232	119.9
C133—C132—C131	120.1 (2)	C231—C236—C235	121.0 (3)
C133—C132—H132	119.9	C231—C236—H236	119.5
C131—C132—H132	119.9	C235—C236—H236	119.5
C226—C221—C222	119.1 (2)	C224—C223—C222	120.4 (3)
C226—C221—P2	122.9 (2)	C224—C223—H223	119.8
C222—C221—P2	117.9 (2)	C222—C223—H223	119.8
C215—C216—C211	120.7 (3)	C234—C235—C236	119.3 (3)
C215—C216—H216	119.6	C234—C235—H235	120.3
C211—C216—H216	119.6	C236—C235—H235	120.3
C123—C122—C121	121.4 (3)	C334—C335—C336	120.2 (3)
C123—C122—H122	119.3	C334—C335—H335	119.9
C121—C122—H122	119.3	C336—C335—H335	119.9
C313—C312—C311	120.3 (3)	C234—C233—C232	120.0 (3)
C313—C312—H312	119.8	C234—C233—H233	120.0
C311—C312—H312	119.8	C232—C233—H233	120.0
C224—C225—C226	120.1 (3)	C315—C314—C313	120.0 (3)
C224—C225—H225	119.9	C315—C314—H314	120.0
C226—C225—H225	119.9	C313—C314—H314	120.0
C211—C212—C213	120.9 (2)	C233—C234—C235	120.6 (3)
C211—C212—H212	119.6	C233—C234—H234	119.7
C213—C212—H212	119.6	C235—C234—H234	119.7
C122—C121—C126	118.3 (2)	C314—C315—C316	120.5 (3)
C122—C121—P1	118.44 (18)	C314—C315—H315	119.7
C126—C121—P1	123.22 (19)	C316—C315—H315	119.7
C325—C326—C321	120.1 (3)	C01—O01—H01	109.5
C325—C326—H326	120.0	O01—C01—H01A	109.5
C321—C326—H326	120.0	O01—C01—H01B	109.5
C115—C116—C111	119.8 (2)	H01A—C01—H01B	109.5
C115—C116—H116	120.1	O01—C01—H01C	109.5
C111—C116—H116	120.1	H01A—C01—H01C	109.5
C114—C113—C112	120.1 (2)	H01B—C01—H01C	109.5
C114—C113—H113	119.9		
			
O1—Cu—P1—C111	−44.63 (10)	Cu—P3—C331—C336	147.10 (19)
P2—Cu—P1—C111	−164.03 (8)	C136—C131—C132—C133	−1.2 (4)
P3—Cu—P1—C111	54.58 (9)	P1—C131—C132—C133	178.7 (2)
O1—Cu—P1—C121	−164.54 (10)	C231—P2—C221—C226	41.8 (2)
P2—Cu—P1—C121	76.06 (9)	C211—P2—C221—C226	−63.8 (2)
P3—Cu—P1—C121	−65.34 (9)	Cu—P2—C221—C226	170.72 (19)
O1—Cu—P1—C131	73.89 (10)	C231—P2—C221—C222	−141.0 (2)
P2—Cu—P1—C131	−45.51 (9)	C211—P2—C221—C222	113.3 (2)
P3—Cu—P1—C131	173.09 (9)	Cu—P2—C221—C222	−12.1 (2)
O1—Cu—P2—C231	79.69 (11)	C212—C211—C216—C215	1.5 (4)
P3—Cu—P2—C231	−25.50 (10)	P2—C211—C216—C215	−176.5 (2)
P1—Cu—P2—C231	−166.70 (9)	C316—C311—C312—C313	0.4 (4)
O1—Cu—P2—C221	−44.25 (11)	P3—C311—C312—C313	178.0 (2)
P3—Cu—P2—C221	−149.45 (9)	C223—C224—C225—C226	0.8 (5)
P1—Cu—P2—C221	69.35 (10)	C216—C211—C212—C213	−0.6 (4)
O1—Cu—P2—C211	−163.59 (10)	P2—C211—C212—C213	177.49 (19)
P3—Cu—P2—C211	91.21 (8)	C123—C122—C121—C126	0.8 (4)
P1—Cu—P2—C211	−49.98 (9)	C123—C122—C121—P1	−177.7 (2)
O1—Cu—P3—C331	−159.83 (10)	C111—P1—C121—C122	−76.8 (2)
P2—Cu—P3—C331	−45.58 (9)	C131—P1—C121—C122	178.80 (19)
P1—Cu—P3—C331	98.95 (9)	Cu—P1—C121—C122	49.6 (2)
O1—Cu—P3—C311	−37.52 (11)	C111—P1—C121—C126	104.8 (2)
P2—Cu—P3—C311	76.73 (10)	C131—P1—C121—C126	0.4 (2)
P1—Cu—P3—C311	−138.73 (10)	Cu—P1—C121—C126	−128.8 (2)
O1—Cu—P3—C321	80.52 (10)	C322—C321—C326—C325	0.7 (4)
P2—Cu—P3—C321	−165.23 (9)	P3—C321—C326—C325	−179.7 (2)
P1—Cu—P3—C321	−20.69 (10)	C114—C115—C116—C111	−0.2 (4)
P2—Cu—O1—N	−27.8 (2)	C112—C111—C116—C115	1.6 (4)
P3—Cu—O1—N	89.75 (19)	P1—C111—C116—C115	−172.9 (2)
P1—Cu—O1—N	−155.85 (18)	C111—C112—C113—C114	−1.0 (4)
Cu—O1—N—O3	19.2 (3)	C131—C132—C133—C134	0.0 (4)
Cu—O1—N—O2	−161.71 (18)	C226—C221—C222—C223	2.7 (4)
C121—P1—C111—C116	−10.8 (2)	P2—C221—C222—C223	−174.5 (2)
C131—P1—C111—C116	95.2 (2)	C123—C124—C125—C126	0.9 (5)
Cu—P1—C111—C116	−137.20 (19)	C132—C131—C136—C135	1.2 (4)
C121—P1—C111—C112	174.68 (18)	P1—C131—C136—C135	−178.7 (2)
C131—P1—C111—C112	−79.3 (2)	C116—C115—C114—C113	−1.8 (5)
Cu—P1—C111—C112	48.3 (2)	C112—C113—C114—C115	2.4 (4)
C221—P2—C231—C236	−154.1 (2)	C326—C321—C322—C323	0.1 (4)
C211—P2—C231—C236	−48.3 (2)	P3—C321—C322—C323	−179.4 (2)
Cu—P2—C231—C236	75.2 (2)	C325—C324—C323—C322	1.9 (4)
C221—P2—C231—C232	33.1 (2)	C321—C322—C323—C324	−1.4 (4)
C211—P2—C231—C232	138.9 (2)	C211—C216—C215—C214	−1.2 (4)
Cu—P2—C231—C232	−97.6 (2)	C132—C133—C134—C135	1.0 (4)
C331—P3—C321—C322	−5.1 (2)	C133—C134—C135—C136	−1.0 (4)
C311—P3—C321—C322	−111.8 (2)	C131—C136—C135—C134	−0.1 (4)
Cu—P3—C321—C322	121.6 (2)	C216—C215—C214—C213	−0.1 (4)
C331—P3—C321—C326	175.31 (19)	C331—C332—C333—C334	−1.5 (4)
C311—P3—C321—C326	68.6 (2)	C335—C334—C333—C332	1.2 (4)
Cu—P3—C321—C326	−57.9 (2)	C215—C214—C213—C212	1.1 (4)
C111—P1—C131—C136	173.54 (19)	C211—C212—C213—C214	−0.7 (4)
C121—P1—C131—C136	−80.2 (2)	C124—C125—C126—C121	−0.7 (5)
Cu—P1—C131—C136	47.6 (2)	C122—C121—C126—C125	−0.2 (4)
C111—P1—C131—C132	−6.3 (2)	P1—C121—C126—C125	178.2 (2)
C121—P1—C131—C132	99.9 (2)	C125—C124—C123—C122	−0.2 (5)
Cu—P1—C131—C132	−132.27 (19)	C121—C122—C123—C124	−0.6 (4)
C315—C316—C311—C312	0.1 (5)	C332—C331—C336—C335	0.8 (4)
C315—C316—C311—P3	−177.6 (3)	P3—C331—C336—C335	179.1 (2)
C331—P3—C311—C316	85.7 (2)	C222—C221—C226—C225	−1.5 (4)
C321—P3—C311—C316	−167.7 (2)	P2—C221—C226—C225	175.6 (2)
Cu—P3—C311—C316	−43.0 (3)	C224—C225—C226—C221	−0.3 (5)
C331—P3—C311—C312	−91.8 (2)	C321—C326—C325—C324	−0.2 (4)
C321—P3—C311—C312	14.8 (2)	C323—C324—C325—C326	−1.1 (4)
Cu—P3—C311—C312	139.4 (2)	C311—C312—C313—C314	−0.4 (5)
C116—C111—C112—C113	−1.0 (4)	C236—C231—C232—C233	1.0 (4)
P1—C111—C112—C113	173.72 (19)	P2—C231—C232—C233	173.8 (2)
C231—P2—C211—C212	136.60 (19)	C232—C231—C236—C235	−1.2 (4)
C221—P2—C211—C212	−115.6 (2)	P2—C231—C236—C235	−174.3 (2)
Cu—P2—C211—C212	12.5 (2)	C225—C224—C223—C222	0.4 (5)
C231—P2—C211—C216	−45.4 (2)	C221—C222—C223—C224	−2.2 (5)
C221—P2—C211—C216	62.4 (2)	C231—C236—C235—C234	1.0 (5)
Cu—P2—C211—C216	−169.52 (18)	C333—C334—C335—C336	0.1 (4)
C333—C332—C331—C336	0.6 (4)	C331—C336—C335—C334	−1.1 (4)
C333—C332—C331—P3	−177.87 (19)	C231—C232—C233—C234	−0.6 (4)
C311—P3—C331—C332	−163.81 (19)	C312—C313—C314—C315	0.0 (5)
C321—P3—C331—C332	90.9 (2)	C232—C233—C234—C235	0.3 (5)
Cu—P3—C331—C332	−34.6 (2)	C236—C235—C234—C233	−0.5 (5)
C311—P3—C331—C336	17.8 (2)	C313—C314—C315—C316	0.5 (5)
C321—P3—C331—C336	−87.5 (2)	C311—C316—C315—C314	−0.5 (5)

### Hydrogen-bond geometry (Å, °)

**Table:**

*D*—H···*A*	*D*—H	H···*A*	*D*···*A*	*D*—H···*A*
O01—H01···O2	0.84	2.03	2.835 (3)	159
